# Effectiveness of a multifaceted implementation strategy for improving adherence to the guideline for prevention of mental ill-health among school personnel in Sweden: a cluster randomized trial

**DOI:** 10.1186/s13012-022-01196-6

**Published:** 2022-03-12

**Authors:** Anna Toropova, Christina Björklund, Gunnar Bergström, Liselotte Schäfer Elinder, Kjerstin Stigmar, Charlotte Wåhlin, Irene Jensen, Lydia Kwak

**Affiliations:** 1 Unit of Intervention and Implementation Research for Worker Health, Institute for Environmental Medicine, Karolinska Institute, 171 77 Stockholm, Sweden; 2 Department of Occupational Health Sciences and Psychology, Centre for Musculoskeletal Research, University of Gävle, 801 76 Gävle, Sweden; 3 Department of Global Public Health, Karolinska Institute, 171 77 Stockholm, Sweden; 4 Centre for Epidemiology and Community Medicine, Region Stockholm, 104 31 Stockholm, Sweden; 5 Department of Health Sciences, Lund University, 221 00 Lund, Sweden; 6 Skåne University Hospital, 221 85 Lund, Sweden; 7 Occupational and Environmental Medicine Centre, Department of Health, Medicine and Caring Sciences, Division of Prevention, Rehabilitation and Community Medicine, Linköping University, 581 85 Linköping, Sweden

**Keywords:** Implementation strategy, Mental health, Schools, Randomized controlled trial, Adherence to guideline recommendations

## Abstract

**Background:**

There is limited research on prevention of mental ill-health of school personnel and the systematic management of school work environments. The aim of this study was to assess the effectiveness of implementing the guideline recommendations for the prevention of mental ill-health in schools, in particular, whether there was a difference in adherence to guideline recommendations between a multifaceted (group 1) and single implementation strategy (group 2) from baseline to 6 and to 12 months.

**Method:**

We conducted a cluster-randomized controlled trial with a 6- and 12-month follow-up. Data was collected from nearly 700 participants in 19 Swedish schools. Participants were school personnel working under the management of a school principal. The single implementation strategy consisted of one educational meeting, while the multifaceted implementation strategy comprised an educational meeting, an ongoing training in the form of workshops, implementation teams and Plan-Do-Study-Act cycles. Adherence was measured with a self-reported questionnaire. Generalized Linear Mixed Models were used to assess the difference between groups in adherence to the guideline between baseline, 6-, and 12-months follow-up.

**Results:**

There were no statistically significant differences between the groups in improvements in adherence to the guideline between baseline, 6-, and 12-months follow-up. However, among those schools that did not undergo any organizational changes during the 12 months of the study significant differences between groups were observed at 12 months for one of the indicators.

**Conclusions:**

The multifaceted strategy was no more effective than the single strategy in improving guideline adherence. There are some limitations to the study, such as the measurement of the implementation outcome measure of adherence. The outcome measure was developed in a systematic manner by the research team, assessing specific target behaviors relevant to the guideline recommendations, however not psychometrically tested, which warrants a careful interpretation of the results.

**Trial registration:**

ClinicalTrials.gov, 150571. Registered 12 September 2017.

**Supplementary Information:**

The online version contains supplementary material available at 10.1186/s13012-022-01196-6.

Contributions to the literature

- Findings from this study contribute with knowledge on the effectiveness of two different implementation strategies on adherence to guidelines in the school setting
- Results demonstrate the importance of taking into consideration important school contextual factors, such as organizational stability and leadership
- This study fills the identified research gaps in the context of school-based implementation research in general and in the Swedish school context in particular

## Introduction

The school work environment, in Sweden and internationally, is known for a number of organizational and social work-related risk factors for common mental disorders [1–4]. Failure to properly address these factors has been shown to lead to the increased likelihood of mental ill-health and sick-leave absence among school personnel [1, 5]. The high prevalence of mental ill-health and related sick-leave observed among school personnel in Sweden underscores the urgent need to prevent mental ill-health among these occupational groups [6]. One way to prevent mental ill-health at the workplace is through the management of organizational and social work-related risk factors [7].

In 2015, new provisions about organizational and social work environment were introduced in Sweden by the Swedish Work Environment Authority with the purpose to promote a good work environment and prevent risks of ill health due to organizational and social conditions in the work environment [8]. In short, these provisions contain rules concerning work environment policy and knowledge requirements and require the employer to regularly investigate and assess what risks may arise and to take corrective measures to manage these risks [8]. Parallel with these new provisions a national evidence-based occupational health guideline was launched to support employers with the prevention of mental ill-health within their organization [9]. The guideline describes a working model for the systematic prevention of mental ill-health at the workplace and is a practical complement to the provisions [9].

Even though guidelines and regulations are an essential part of achieving sustainable working environments, research has shown that the mere existence of guidelines and regulations does not guarantee their use in practice [10]. A national school assessment report from the Swedish Work Environment Authority, showed that most of the inspected schools did not take adequate actions towards preventing organizational and social risks in the work environment [11]. In particular, the report identified the following aspects lacking in the inspected schools: (a) a written assessment of the risks found in the work environment; (b) written documentation on how systematic actions on improving the work environment should be carried out; (c) written action plans for upcoming measures; and (d) causes and risk-assessment of unhealthy workload levels. Since these aspects form the essence of the organizational and social risk management to prevent work-related mental ill-health [9, 12], the need to support schools in filling the gap is evident. One way of doing this is for schools to implement the guideline for mental ill-health within their workplace. As several studies have shown that solely disseminating guidelines and regulations does not result in full implementation in practice, additional implementation strategies are needed to support schools in the implementation process [10].

To support schools with the implementation of the guideline we conducted a cluster-randomized waiting-list controlled trial between 2017 and 2019 with the primary objective to compare the effectiveness of two different types of strategies for implementing the guideline within schools [13]. Specifically, a multifaceted implementation strategy containing an educational meeting, ongoing training operationalized as five workshops, local implementation teams and an iterative and evaluative strategy (Plan-Do-Study-Act) was compared with a single strategy containing only the educational meeting. The strategies were systematically developed, theory-based, and targeted barriers and facilitators prospectively identified during the planning workshops with school principals [13]. The model used was the well-established implementation model COM-B developed by Michie et al., which posits that behavior is a function of three components: Capability (C), Opportunity (O), and Motivation (M) [14]. Capability refers to the ability to engage in the thought or physical processes necessary for the behavior, e.g., knowledge and skills. Motivation refers to those brain processes that direct behavior, and include reflective and automatic motivation, e.g., analytical decision-making and emotional responses. Opportunity refers to those factors that lie outside the individual that influence behavior, e.g., social support and prompts. To change behavior, for example behaviors related to implementing a guideline, one has to have the capability, opportunity, and motivation to do so. In accordance with previous studies, it was hypothesized that the single implementation strategy, in this case education about the guideline, would be less effective in increasing adherence to the guideline and likely only have an impact on capability (e.g., an increase in knowledge of the guideline) [15]. Based on the COM-B model [14], it was further hypothesized that the multifaceted implementation strategy would have an additional effect beyond the single strategy by targeting capability, opportunity, and motivation to implement the guideline, contributing to the development of motivation, social support etc., which in turn would increase adherence to the guideline.

This study reports the results of the outcome-evaluation of the cluster-randomized waiting-list controlled trial. More specifically, the research question is to evaluate whether there is a difference in the outcome measure of adherence to guideline recommendations between the multifaceted and single implementation strategy from baseline to 6 and 12 months.

## Methods

### Trial design

The project was a cluster-randomized wait-list controlled trial with parallel groups conducted between 2017 and 2019. Randomization was carried out at the school level, where schools were allocated to group 1 (multifaceted strategy; intervention group) or group 2 (single strategy; wait-list minimal strategy control group) on a 1:1 ratio [13]. Schools in group 1 were offered the multifaceted implementation strategy (comprising a day’s educational meeting, formation of an implementation team, five workshops and an iterative and evaluative strategy (Plan-Do-Study-Act cycles) within 12 months. Schools in group 2 were offered the educational meeting strategy only. After 12 months, group 2 schools were offered the other three implementation strategies [13].

### Context

The Swedish school system comprises pre-school, compulsory school, and upper secondary school. Children can attend a voluntary pre-school until the age of 6 when they start compulsory school. Compulsory schooling is until grade 9, after which students can choose to continue a 3-year upper secondary school program [16]. The school year lasts from around mid-August to around mid-June. Schools are tax-financed and may be either private or public. The municipality organizes and allocates resources to all schools. Principals and teachers are responsible for students achieving national educational standards and goals. Principals are also responsible for providing a safe work environment for their students and personnel.

### Targeted sites and population

The project was carried out in 19 compulsory public schools in two Swedish municipalities. Overall, the municipalities featured distinctive geographical characteristics, with one municipality situated in an urban area and another in a rural area. Schools were recruited in a two-step process. In step 1, advertisements were placed in newsletter, for e.g., the Swedish Union of Teachers and The Swedish Association of School Principals, social media channels, as well as university webpage and work environment blog. Next, interested municipalities and their schools’ principals were given a detailed oral presentation about the project.

**Fig. 1:**
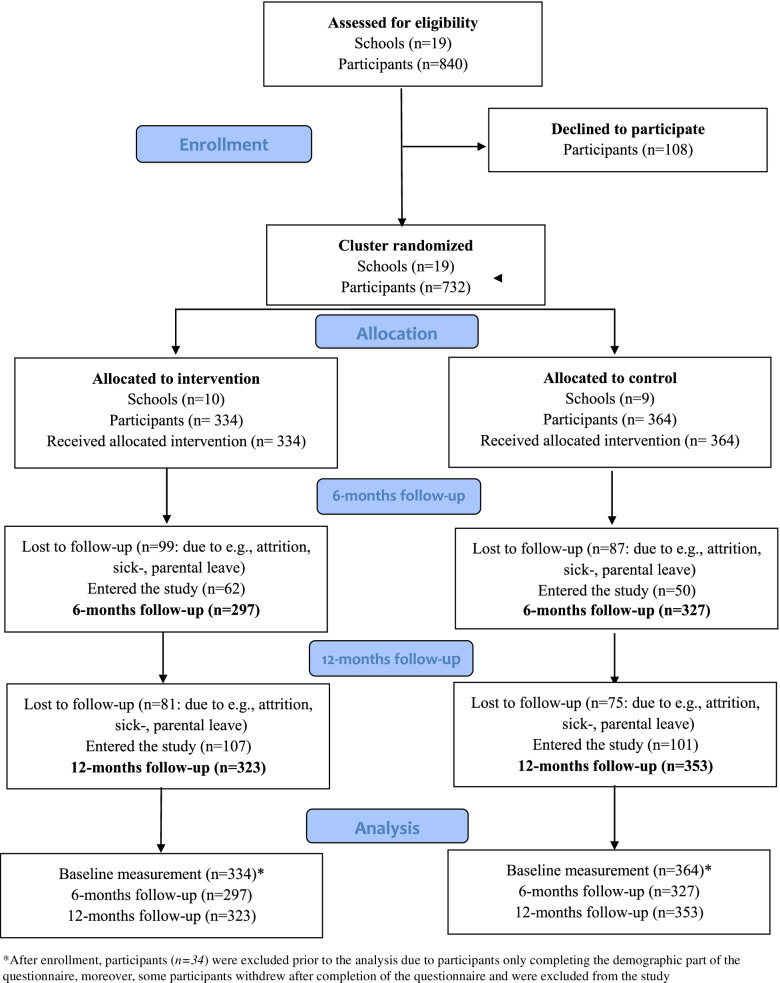
Consort flow diagram for the cluster-randomized study

All employees of the participating schools—teachers, school administrators, recreational pedagogues etc.,—840 individuals in total—were invited to participate in the trial and complete the baseline questionnaire from September to October 2017. The exclusion criteria for participation in the evaluation were not being employed by the school, such as cleaning staff. Study participants were recruited by various means, presented in detail in the study protocol [13]. A total of 698 individuals answered the questionnaire. The total response rate was 83.1%. The study sample for this study was all school personnel that completed an outcome questionnaire at baseline, 6, and/or 12 months. Due to high personnel-turnover, an open cohort was employed, where participants could join at 6 and/or 12 months (Fig. 1).

In this study, the outcome evaluation of the school principals is not reported, which is a deviation from the protocol [13]. Due to the different nature of questions used to assess adherence among school principals and school personnel [13], only the results of the school personnel’s participation are used. Besides, we see employee-reported adherence as a less biased implementation outcome as compared to the school principals’ adherence, which in our opinion is more prone to be affected by social desirability.

### Description of the intervention

The intervention was the *guideline for prevention of mental ill-health at the workplace* [9]. The guideline consists of two sections. The first section gives recommendations on how to prevent work-related mental ill-health from an organizational perspective and is directed towards employer’s systematic occupational, safety, and health work. The second section gives recommendations on the treatment of mental ill-health and is directed at occupational health professionals. The focus of the present trial was on supporting schools with the implementation of the first section of the guideline, which recommends an organizational approach to the prevention of mental ill-health. The organizational approach recommended in the guideline includes the involvement of all school personnel. School personnel are, for example, recommended to be involved in the prioritization of work environment risk factors that need changing in order to prevent mental ill-health. The guideline recommends that this is done in group-discussions with all school personnel; accordingly, action-plans should be developed together with school personnel describing changes that need to be made. Another recommendation relates to how well documents describing work environment routines are established and known among school personnel [9]. Every guideline recommendation manifests itself in several specific target behaviors, defined by the research team. Targeting those behaviors was hypothesized to lead to a desired change. An overview of the guideline recommendations is presented in Table 1.

**Table 1:** Guideline recommendations

**Recommendation**	
1. Workplaces should have well-established policies related to the social and organizational risk management	
2.Employers have knowledge on the relationship between social and organizational risks and mental ill-health	
3.Workplaces regularly assess their social and organizational work environment and intervene on identified social and organizational risk factors	

Kwak et al .[13] and Jensen et al. [9]

### Description of the implementation strategies

Implementation strategies were chosen based on existing taxonomies [17] targeting pre-identified barriers and facilitators. Barriers and facilitators were identified from the Swedish school context during planning workshops with the school-principals [13] and from a European assessment of barriers and facilitators for managing social and organizational risks at the workplace [18]. In collaboration with the school-principals and expert-group, implementation strategies were matched to the identified barriers and facilitators. Table 2 summarizes the results of workshop discussions on the knowledge, attitudes, and behaviors for ensuring guideline adherence as well as identified barriers and facilitators. The content of the strategies was further driven by specific behavioral aspects derived from the COM-B model [14]. Matching of the strategies to the relevant COM-B constructs is exemplified in Table 3.

**Table 2:** Barriers and facilitators prospectively identified on the basis of the literature and the planning workshops with the principals

Barriers	Facilitators
*Capability*	
Limited knowledge on the social and organizational risk management	Knowledge of the guideline recommendations
*Opportunity*	
Limited support	Collegial and organizational social support
*Motivation*	
Lack of time	Developing the right attitude
Unable to prioritize	Motivation, enthusiasm, engagement
Unable to carry out plans	Systematic and structural approach
Vague professional role	Clarity

**Table 3:** Some examples of matching implementation strategies to COM-B constructs

Implementation strategies	Content	COM-B
Educational meeting	The purpose and the content of the guideline is introduced by the research team	Psychological capability
	Exercise during which schools reflect whether the recommendations need adjustment to the school context	Reflective motivation
Implementation team	Teams demonstrate social support and modelling, and experience social comparison	Social opportunity
The *Plan-Do-Study Act* cycles	Implementation of the guideline’s recommendations adopts a structured approach	Reflective motivation
Workshop series	The notion of SMART-goals is introduced by the research team	Psychological capability
	Exercise where the implementation team outlines a SMART-goal for a recommendation to be implemented	Reflective motivation
	Plenary discussion on how the implementation teams and the municipality’s educational board can provide support and communicate	Social opportunity
	Implementation teams present results of their plan’s execution, encountered barriers and facilitators, and necessary adaptations to the plan	Reflective motivation

A brief description of the educational meeting, implementation teams, workshops and Plan-Do-Study-Act improvement cycles is given below and in Table 4 (see also TIDieR checklist, Additional file 1). For a thorough description see the study protocol [13].

**Table 4:** Description of the single implementation strategy and multifaceted implementation strategy

	Multifaceted implementation strategy			
	Single implementation strategy			
	Educational meeting	Implementation team	Workshops	Plan-Do-Study-Act cycles
Rationale, theory, or goal of the elements essential to the strategy	COM-B constructs: capability, opportunity, motivation. The meeting was aimed at providing participants with knowledge and skills related to the guideline, barriers, and facilitators. Schools made an action-plan for the implementation of a guideline recommendation of choice.	COM-B constructs: opportunity. The rationale for forming an implementation team at each school was that school principals had indicated to need support from personnel with implementing the guideline.	COM-B constructs: capability, opportunity, motivation. The workshops were aimed at providing teams with knowledge and skills regarding (1) the recommendations of the guideline, (2) implementation processes, and (3) Plan-Do-Study-Act cycles.	COM-B constructs: capability, opportunity, motivation. The goal of this iterative and evaluative strategy was for teams to identify the needed change, facilitate the change, assess its success, and adapt to the change based on feedback to arrive at targeted solutions.
Materials, procedures, activities, and/or processes used	Materials: the guideline and a compendium, which included handouts of the presentations and documents related to exercises. Procedure: the meeting included PowerPoint presentations, plenary discussions, and five different group-exercises.	Materials: school principals received instructions, including a template to support them with forming their implementation team. Procedure: teams participated in workshops, conducted Plan-Do-Study-Act cycles, and met between workshops.	Materials: handouts of the presentations and documents related to exercises. Procedure: workshops included presentations on the guideline, implementation processes and Plan-Do-Study-Act cycles, plenary discussions, and group exercises.	Materials: exercises related to forming SMART-goals, templates of Plan-Do-Study-Act cycles were made for the purpose of the study. Procedure: PDSA-cycles were performed during and between workshops by the implementation teams
Strategy provider’s expertise and background	The meeting was held by an implementation expert and licensed occupational health psychologist	Implementation expert sent instructions by mail to the school principal. School-principal selected team-members, e.g., health and safety officer, union representative etc.	Implementation expert and licensed psychologist with occupational health expertise gave workshops 1–3; the same implementation expert and a researcher with expertise in the guideline recommendations gave workshops 4–5.	Implementation expert introduced the methodology of Plan-Do-Study-Act cycles during the first workshop and supported teams with their cycles during proceeding workshops.
Modes of delivery of the strategy provided individually or in a group.	The meeting was delivered at each municipality face-to-face to all public compulsory schools within that municipality.	Individual instructions sent to school-principals by mail.	The workshops were delivered at each municipality face-to-face to all implementation teams within that municipality.	Introduced face-to-face to all implementation teams during workshop 1.
Type of location where the strategy occurred, including necessary infrastructure	At one of the municipalities the meeting was conducted at the nearby university and at the other municipality at the city hall. Infrastructure included a projector for presentations, tables set up for group-work.	Implementation teams met at each workshop and at their own school between workshops.	At one of the municipalities the workshops were given at one of the participating schools and at the other at the city hall. Infrastructure included a projector for presentations, tables set up for group-work.	The first cycle was started during workshop 1. Cycles continued between and during the remaining workshops.
Number of times the strategy was delivered, period including the number of sessions, schedule, and duration.	One educational meeting was given at each municipality in October 2017. The meeting was given between 9.00 and 16.15.	Teams were formed per school in October 2017 prior to workshop 1. Teams were intended to last the whole study period and preferably beyond.	Five workshops (2.5 h per workshop) were given at each municipality between October 2017 and June 2018.	The number of cycles conducted varied between implementation teams. No instructions were given for a minimum or maximum number of cycles to be conducted.
Personalization of the strategy (what, why, when, and how).	The schedule, presentations and exercises of the educational meeting were the same at each municipality.	Team-members were personally selected by the school principal following instructions given by the implementation expert.	The schedule and exercises of the workshops were the same at each municipality.	Each team conducted personalized Plan-Do-Study-Act cycles adapted to their local needs. Personalization was conducted by the teams
Modification of the strategy during the study - changes made (what, why, when, and how).	No modifications were made	No modifications were made.	No modifications were made	No modifications were made
Planned: Assessment of strategy adherence or fidelity (how, by whom), strategies used to maintain or improve fidelity. Actual: If strategy adherence or fidelity was assessed, was the strategy delivered as planned.	Fidelity to the educational meeting was assessed by checklist. Overall, the educational meeting was delivered as intended.	The research-team assessed whether each school formed an implementation team. Implementation teams were formed for each school. Teams were instructed to make a communication plan on how to communicate with their municipality. This was executed in one municipality.	Fidelity to workshops was assessed by checklist during the workshops by the research-team. Overall, the workshops were delivered as intended. With exception of one of the exercises of workshop 3, which was not fully delivered as intended in one municipality. Moreover, the municipality did not participate in workshop 2 in one municipality.	Fidelity to the Plan-Do-Study-Act cycles was assessed by letting teams present their progress during the start of workshop 2–5. This was delivered as planned.

### Educational meeting

The goal of the meeting was to provide participants with knowledge and skills of using the guideline and to support schools with making an action-plan for the implementation of a guideline recommendation of choice. The meeting included PowerPoint presentations, plenary discussions and five different group-exercises. The meeting targeted COM-B constructs: capability, opportunity, and motivation. At the start of the meeting participants received the guideline and a compendium, which included handouts of the presentations and documents related to the exercises. The meeting was held face-to-face at each municipality by an implementation expert and a licensed occupational health psychologist. At one municipality the meeting was given at a nearby university, at the other municipality it was held at the city hall. At both municipalities the meeting was given in October 2017. Participants of the meeting were implementation teams of 4–6 individuals (school principal, in addition to for example teacher union representatives, teacher representatives, and health and safety officers, assistant school principals) per school.

### Implementation teams

The rationale for forming an implementation team at each school was that school principals had indicated during planning workshops that they required support from personnel with implementing the guideline. The teams mainly targeted the COM-B construct opportunity (i.e., social support). Prior to the educational meeting (in October 2017), school principals received instructions, including a template, to help them identify which employees would be best suited to be members of the team. In the template, school-principals indicated the role of each member and a motivation for inclusion. School principals also described the support members needed to fulfill their role, including resources such as time. The team’s main task was to lead the guideline implementation in their schools and conduct the Plan-Do-Study-Act cycles [19]. An additional task of the team was facilitating communication with the municipality to receive support during their implementation process. Each team comprised of 4–6 participants. All teams included a school principal, often in combination with an assistant school-principal, a health and safety officer and a teacher union representative.

### Ongoing training through workshop-series

The aim of the workshop series was to provide the implementation teams with knowledge and skills regarding (1) the recommendations of the guideline, (2) implementation processes, and (3) Plan-Do-Study-Act cycles. The workshops included presentations on the recommendations of the guideline, implementation processes (including barriers and facilitators), and on the methodology related to Plan-Do-Study-Act cycles, plenary discussions, and group exercises. During the workshops, participants received handouts of the presentations and documents related to exercises. An implementation expert and a licensed psychologist with occupational health expertise gave workshops 1–3; the same implementation expert and a researcher with expertise in the guideline recommendations gave workshops 4–5. Five workshops (2.5 h per workshop) were given face-to-face to all implementation teams at each municipality between October 2017 and June 2018.

### Plan-Do-Study-Act improvement cycles

The goal of this iterative and evaluative strategy was for implementation teams to identify the needed change, facilitate the change, assess its success, and adapt to the change based on feedback to arrive at targeted solutions. Plan-Do-Study-Act cycles have shown its suitability for use in complex systems [19], such as schools. The methodology was presented during the first workshop by the implementation expert by using a PowerPoint presentation. During workshop 1 implementation, teams conducted an exercise aimed at developing a SMART-goal to help them formulate their Plan. Moreover, teams received materials in which they could document their Plan-Do-Study-Act cycles and progress, including barriers and facilitators. Teams were instructed to plan for change (Plan) during the workshops and implement the planned change (Do) during workshops. The progress (Study) was presented plenary by the teams at the start of each workshop. During the proceeding workshop teams accordingly adapted, if needed, their plan (Act), and implemented the plan (Do) until the next workshop. The number of cycles differed by team; the first cycle started in October 2017.

### Data collection and outcomes

#### Questionnaire

A questionnaire was developed by the research team [13] covering participants’ socio-demographic characteristics, such as age, gender, education level, years of working experience in the field and in the current school, and their professional title. The primary outcome of this study was adherence to the three guideline recommendations at 6 and 12 months. It was assessed based on self-reported outcome measures by the school personnel in the participating schools. In other words, whether school personnel participated in any changes in their school’s management of organizational and social work-related risk factors as recommended by the guideline. Guideline adherence was assessed with 8 items (indicators) reflecting the guideline recommendations. Response options included Likert-scale items ranging from ‘strongly disagree’ to ‘strongly agree’, as well as ‘neither disagree nor agree’ and ‘I do not know’ response options. The questions concerning recommendation 3 were precluded by the question: “When did your school or school principal conducted the latest assessment of the employees’ work environment and mental health?”. Response options for this question were ‘0–6’, ‘7–12’, ‘13–18’, and ‘19–24’ months, ‘No assessment was conducted” and ‘I do not remember’. A comprehensive description of the adherence indicators pertaining to each of the guideline recommendations is provided in Table 5. The questionnaire was administered at baseline, 6-, and 12 months during the school’s staff meeting, when school personnel filled in paper questionnaires and returned them to the research team. Those participants who were absent at the staff meeting had an opportunity to fill in an online survey, with three weekly reminders sent to non-respondents.

**Table 5:** Indicators of the guideline recommendations adherence

Guideline recommendations	Indicators of the guideline recommendations adherence
Recommendation 1	1a. I am familiar with the content of our school’s work environment documents. 1b. I act in accordance with our school’s work environment documents.
Recommendation 2	2a. I notice that my immediate leadership has the knowledge on how work environment affects employee’s mental health
Recommendation 3	3a. During the latest assessment the results were communicated to us employees by someone in a leadership position 3b. During the latest assessment I was given an opportunity as an employee to participate in the discussion of the results 3c. During the latest assessment we did a joint planning of measures based on the results 3d. During the latest assessment we created an action plan for the measures to be taken 3e. During the latest assessment we used the action plan to monitor the implementation of the planned measures

Kwak et al .[13] and Jensen et al. [9]

#### School organizational change

Information on changes occurring within the school’s organization was collected from all schools as it was hypothesized that these changes would likely impact the implementation process. The information was collected during the meetings with municipalities as well as via telephone interviews with the principals in all participating schools on the basis of a semi-structured interview guide. The interview concerned any sort of organizational change taking place in a school, such as principal turnover and/or school restructuring. Additionally, observations by the research team were systematically conducted on the basis of notes and logbooks.

### Ethical approval

The trial was approved by the Regional Ethical Board in Stockholm (nr. 2017/984-31/5). The study participants were thoroughly informed about the nature of the study, the voluntary type of participation, confidentiality of the responses, and a possibility to withdraw from the study at any time. Thereafter, informed consent was collected from the study participants.

### Statistical analysis

Results of the analysis are reported according to the CONSORT guideline for pragmatic and cluster randomized trials (see CONSORT checklist, Additional file 2). Participants’ responses on the adherence indicators to each of the guideline recommendations were dichotomized into ‘adherence’ (those responding, ‘strongly agree’ and ‘agree’ to guideline adherence indicators) and ‘non-adherence’ (those responding ‘strongly disagree’, ‘disagree’, ‘neither agree nor disagree’ or ‘I do not know’). Adherence was coded as ‘1’, while non-adherence was coded as ‘0’.

The number of missing values was under 10% and the inspection of missing values did not reveal any systematic pattern. Therefore, no additional adjustment was performed.

First, absolute changes in adherence between baseline and 6 and 12 months (within group) in adherence were calculated and classified according to the following pre-defined classification: small (< 5%); modest (between 5 and 10%), moderate (between 11 and 20%), or large (> 20%) [20].

Logistic generalized linear mixed regression modelling (GLMM) was employed to examine a change in adherence to guideline recommendations from baseline to 6 and 12 months in group 1 compared to group 2 (between group). The nested data structure was accounted for with a random intercept by using a person-specific random intercept to model the within-subject clustering over time, and a school specific random intercept to model clustering within schools.

The binary outcome of adherence vs. non-adherence at baseline, 6, and 12 months was treated as the dependent variable. Group and time variables were treated as fixed factors, while an interaction of group and time was used as an indicator of the intervention effect at the different, discrete time points. The baseline adherence value was included in the model as one of the discrete time points, contrasting the other time points against it. We hypothesized that the length of work experience in the school as well as school organizational change could contribute to differences in adherence to guideline recommendations; therefore, these two variables were used as fixed covariates in the model. Separate models were fit for each of the guideline recommendations.

Two types of sensitivity analysis were performed. The first one included those participants who filled in the questionnaire at all three time points (baseline, 6 months, and 12 months), while the second one included the data for the participants, whose schools did not undergo any organizational changes during the 12 months of the study.

Estimates of treatment effects were presented as odds ratios with 95% confidence intervals. Alpha significance level was set to 0.05 for two-sided statistical tests. In interpreting the results of this study, a particular focus was placed on examining treatment effects along with the corresponding confidence intervals, in order to address both statistical and clinical significance of the results [21, 22].

IBM SPSS Statistics 26 was used to conduct the analyses for this study.

## Results

### Descriptive statistics

Descriptive statistics for the study participants are presented in Table 6.

**Table 6:** Participant characteristics at baseline

School personnel characteristics	Group 1 (***N*** = 336)			Group 2 (***N*** = 362)		
	*N*	%	Mean (SD)	*N*	%	Mean (SD)
**Age**	325		47.26 (11.95)	357		44.84 (11.68)
**Gender (female)**	254	76.5		272	74.9	
**Professional title**	304			343		
Teacher	232	76.3		248	72.3	
Other school personnel (school administrators, recreational pedagogues etc.)	72	23.7		95	27.7	
**Education level**	331			359		
Basic education	9	2.7		10	2.8	
Secondary education	59	17.7		64	17.8	
University education	256	77.3		272	75.8	
Post-graduate education	7	2.1		13	3.6	
**Work experience in the field**	331			362		
Less than 5 years	85	25.7		108	29.8	
5–14 years	94	28.4		115	31.8	
15–24 years	72	21.6		78	21.5	
25–34 years	46	13.8		36	9.9	
35 or more years	34	10.2		25	6.9	
**Work experience in the current school**	322			345		
Less than 5 years	196	60.9		226	65.5	
5–14 years	62	19.3		84	24.3	
15–24 years	45	13.9		27	7.8	
25–34 years	14	4.3		7	2.0	
35 or more years	5	1.5		1	.3	

During the trial period, organizational changes were noted among two schools in group 1 and one school in group 2. At two schools the upper-level of education was transferred to another school, and two schools changed school principal.

### Guideline recommendations adherence

Participant responses to the indicators of guideline recommendations adherence are shown in Table 7.

**Table 7:** Adherence^a^ to guideline recommendations after 6 and 12 months

	Group 1				Group 2			
	*N* (%)			%	*N* (%)			%
	Baseline	6 months	12 months	Absolute change at 6/12 months	Baseline	6 months	12 months	Absolute change at 6/12 months
**Recommendation 1**								
1a. I am familiar with the content of our school’s work environment documents	39/321 (12.1)	56/288 (19.4)	52/314 (16.6)	7.3/4.5	73/354 (20.6)	72/321 (22.4)	69/349 (19.8)	1.8/− 0.8
1b.I act in accordance with our school’s work environment documents.	51/316 (16.1)	54/286 (18.9)	52/312 (16.7)	2.8/0.6	67/352 (19.0)	66/320 (20.6)	76/345 (22.0)	1.6/2
**Recommendation 2**								
2a. I notice that my immediate leadership has the knowledge on how work environment affects employee’s mental health	48/320 (15.0)	56/286 (19.6)	55/312 (17.6)	4.6/2.6	84/353 (23.8)	71/319 (22.3)	98/349 (28.1)	− 1.5/4.3
**Recommendation 3**								
3a. During the latest assessment the results were communicated to us employees by someone in a leadership position	48/113 (42.5)	45/140 (32.1)	50/131 (38.2)	− 10.4/− 4.3	66/136 (48.5)	63/160 (39.4)	97/170 (57.1)	− 9.1/8.6
3b. During the latest assessment I was given as opportunity as an employee to participate in the discussion of the result	41/113 (36.3)	45/140 (32.1)	34/132 (25.8)	− 4.2/− 10.5	59/136 (43.4)	62/161 (38.5)	86/169 (50.9)	− 4.9/7.5
3c. During the latest assessment we did a joint planning of measures on the basis of assessment results	14/111 (12.6)	29/139 (20.9)	24/132 (18.2)	8.3/5.6	36/135 (26.7)	40/158 (25.3)	57/169 (33.7)	− 1.4/7
3d. During the latest assessment we created an action plan for measures to be taken	10/113 (8.8)	18/136 (13.2)	15/132 (11.4)	4.4/2.6	30/134 (22.4)	28/160 (17.5)	38/169 (22.5)	− 4.9/0.1
3e. During the latest assessment we used the action plan to monitor the implementation of the planned measures	5/111 (4.5)	12/136 (8.8)	8/130 (6.2)	4.3/1.7	25/133 (18.8)	23/159 (14.5)	27/169 (16.0)	− 4.3/− 2.8

^a^Participants responding ‘completely agree’ and ‘agree’ to the adherence indicators

At 6 months, group 1 schools increased adherence to six of the eight indicators (absolute changes 2.8 to 8.3) and decreased adherence to two indicators (absolute changes − 10.4 to − 4.2). In group 2 schools, adherence increased for two indicators (absolute changes 1.6 to 1.8) and decreased in six indicators (absolute changes − 9.1 to − 1.4). At 12 months, adherence to the six indicators remained higher in group 1 schools compared to baseline adherence. Absolute changes observed between baseline and 12 months were however smaller than those between baseline and 6 months, except for indicator 3a. In group 2 schools, adherence increased at 12 months for six indicators compared to baseline (absolute changes 0.1 to 7.5) and decreased for two indicators.

Results of the generalized linear mixed modeling are presented in Table 8. An OR > 1 means a higher adherence in group 1 schools, while OR < 1 means a higher adherence in group 2 schools. The OR for the indicator 3b at 12 months was statistically significant and in favor of group 2 schools. No further statistical differences were observed.

**Table 8:** The comparative effectiveness of the multifaceted implementation strategy vs. single strategy on adherence to guideline recommendations

Guideline adherence indicators	Intervention effect 6 months^**a**^(group 1 vs. group 2 AOR^**b**^, (CI)	***p*** value	Intervention effect 12 months^**a**^, (group 1 vs. group 2) AOR, (CI)	***p*** value
1a	1.63 (.846–3.122)	.145	1.70 (.868–3.344)	.122
1b	1.13 (.586–2.171)	.719	1.08 (.554–2.113)	.816
2a	1.69 (.891–3.203)	.108	.792 (.409–1.533)	.488
3a	.813 (.367–1.803)	.610	.540 (.241–1.214)	.136
3b	.874 (.387–1.973)	.745	.355 (.151–.832)	.017
3c	1.95 (.743–5.098)	.175	1.17 (.431–3.165)	.759
3d	1.81 (.588–5.553)	.301	1.33 (.408–4.338)	.636
3e	2.82 (.743–10.665)	.128	1.01 (.193–5.298)	.990

^a^In relation to baseline adherence

^b^Adjusted odds ratios for the length of work experience in the school and school organizational change

### Sensitivity analyses

Table 9 shows the results of the sensitivity analysis including participants who filled in the survey at all measurement points, i.e., baseline, 6, and 12 months. The OR for the indicator 1a at 12 months was statistically significant and in favor of group 1 schools. No further statistical differences were observed.

**Table 9:** Sensitivity analysis of comparative effectiveness—participants filling in the survey at all measurement points (*N* = 401)

Guideline adherence indicators	Intervention effect 6 months^**a**^, (group 1 vs. group 2) AOR^**b**^, (CI)	***p*** value	Intervention effect 12 months^**a**^, (group 1 vs. group 2) AOR^**b**^, (CI)	***p*** value
1a	2.09 (.894–4.890)	.089	2.43 (1.035–5.711)	.041
1b	1.39 (.610–3.148)	.436	1.63 (.714–3.702)	.246
2a	2.21 (.972–5.021)	.059	1.16 (.499–2.694)	.730
3a	.75 (.283–1.967)	.553	.61 (.233–1.577)	.304
3b	.85 (.315–2.301)	.751	.41 (.150–1.124)	.083
3c	2.15 (.651–7.074)	.210	1.30 (.386–4.345)	.675
3d	1.51 (.413–5.483)	.535	.858 (.218–3.377)	.826
3e	1.41 (.320–6.191)	.650	.682 (.120–3.864)	.665

^a^In relation to baseline adherence

^b^Adjusted odds ratios for the length of work experience in the school and school organizational change

Table 10 presents the results of the sensitivity analysis including only those schools which had not undergone organizational change. The sensitivity analysis showed a more positive intervention effect (OR > 1) for indicator 1a at 12 months for group 1 compared to group 2 schools. The other ORs were not statistically significant.

**Table 10:** Sensitivity analysis of comparative effectiveness—participants from the schools characterized by organizational stability

Guideline adherence indicators	Intervention effect 6 months^**a**^ (group1 vs. group 2) AOR^**b**^ (CI)	***p*** value	Intervention effect 12 months^**a**^ (group 1 vs. group 2) AOR^**b**^ (CI)	***p*** value
1a	1.96 (.874–4.375)	.103	3.20 (1.408–7.275)	.006
1b	1.05 (.473–2.330)	.904	1.55 (.698–3.427)	.283
2a	1.87 (.863–4.049)	.112	1.11 (.510–2.414)	.792
3a	1.64 (.614–4.380)	.323	1.15 (.422–3.108)	.790
3b	2.14 (.786–5.808)	.136	1.06 (.373–3.006)	.915
3c	2.46 (.766–7.903)	.130	2.19 (.653–7.347)	.204
3d	1.20 (.284–5.063)	.805	1.32 (.302–5.750)	.713
3e	2.20 (.457–10.561)	.325	1.28 (.213–7.709)	.787

^a^In relation to baseline adherence

^b^Adjusted for the length of work experience in the school only

## Discussion

This study aimed to investigate the effect of a multifaceted implementation strategy versus a single implementation strategy on adherence to guideline recommendations for the prevention of mental ill-health in the Swedish school setting. Our main finding is that we could not confirm our hypothesis that the multifaceted strategy was more effective than the single strategy in improving guideline adherence. In the following sections, we will discuss these findings in a greater detail.

### The potential of the multifaceted implementation strategy to improve adherence to guideline recommendations

Our findings are in line with the literature showing inconclusive evidence for the effect of multifaceted implementation strategies on guideline adherence [23, 24]. A systematic review of reviews on the effectiveness of implementation strategies in health care [25] demonstrated small to modest effects of a multifaceted strategy together with a substantial variation within and between interventions. Other reviews have however not found multifaceted implementation strategies to be effective [23, 26]. Due to the variance in multifaceted strategies analyzed in these reviews, i.e., these strategies containing different components, in addition to the diversity of settings and the majority of studies conducted in health care settings, a more careful comparison with the results of this study is warranted. In this study, support was given in the form of an educational meeting and an ongoing training operationalized through a series of workshops. In addition, implementation teams developed specific implementation plans through PDSA, which was hypothesized to further enhance adherence. Other studies on the use of either workshops, PDSA cycles, or implementation team’s engagement as single implementation strategies [19, 20, 27, 28], have shown their effectiveness in improving guideline adherence.

A first possible explanation for our findings is that the implementation strategies may not have been fully implemented as intended. The process evaluation conducted parallel to the trial [Kwak et al., unpublished observations] showed high fidelity to all components of the multifaceted implementation strategy except for one: forming a communication plan facilitating communication between the schools’ implementation teams and key persons in their municipality. Only in one municipality was such communication successfully facilitated. This could be a critical factor undermining the implementation effectiveness in the municipality by offering less support to the schools [29]. Schools most likely need a more structured support with implementation from their municipality. This support could be given by an internal facilitator who enables implementation teams to implement the guideline recommendations within their school through Plan-Do-Study-Act cycles [30]. Facilitation has been identified as the active ingredient of implementation [27] and shown to enhance the uptake of guidelines in primary care settings [30]. Future studies should explore the possibility of adding an internal facilitator at municipality level to the multifaceted implementation strategy. The facilitator could aid with creating and maintaining a supportive organizational context on both the school and municipality level [28–30], to assist schools with their implementation. Moreover, the internal facilitator could perform monitoring of the implementation, which has been found to be one of the five most important strategies to promote implementation efforts in the school setting [31].

A second potential explanation for our findings is the feasibility of the employed implementation strategies in the school context. A recent study evaluating the importance and feasibility of implementation strategies for schools found that both educational meetings and ongoing training, which in the present study was operationalized as a workshop series, were rated as important and feasible strategies [31]. This arguments for including these components in the multifaceted strategy. These components also received overall high ratings on acceptability, appropriateness, and feasibility in our process evaluation [Kwak et al., unpublished observations]. Organizing school personnel implementation team meetings and conducting cyclical small tests of change, operationalized in the present study as Plan-Do-Study-Act cycles, were rated as important but less feasible strategies in a school setting [31]. Implementation teams have been long recognized as critical to carrying out a successful implementation in general, and in a school setting in particular [29, 32]. Even though school principals in the present study were instructed to identify the resources (i.e., time) team members needed to be able to fulfill their role as members, it is possible that high work-demands frequently experienced by teachers [33, 34] may have hindered their possibility to support school-principals with the implementation of the guideline. Time-constraints have in several previous studies been identified as a common barrier to implementation by teachers [33, 35]. In our process evaluation, no indication was given that work-demands influenced teacher’s involvement in the implementation strategies or implementation process [Kwak et al., unpublished observations].

A third explanation for our findings relates to the larger context in which the study has been conducted. As stated earlier, according to the provision of the Swedish Work Environment Authority, Swedish employers, including school principals, are required by law to regularly investigate and assess the risks of work-related mental ill-health and to take corrective measures to manage these risks. Moreover, school management is recommended to present results to their employees and to give them the possibility to discuss results [11]. Even though these new provisions would not automatically lead to behavior change, it could explain why small improvements in several indicators were observed in both groups during the study period. The observed overall low adherence levels to the guideline recommendations however suggests that the implementation strategies might not have been sufficient to increase adherence and demonstrates the need to examine which additional support is needed to enhance the implementation outcome.

Finally, our findings could be explained by unplanned organizational changes occurring at the school-level. During the first year of the project, two schools in group 1 and one school in group 2 underwent large organizational changes. The changes involved a transfer of the upper-secondary education level to another school at two of the participating schools likely resulting in, among others, re-assignments of personnel, increased uncertainty, and extra workload leading to high stress among school principals and personnel. In addition, at one group 1 school, the school principal resigned 6 months into the project. Results of the sensitivity analyses revealed higher ORs for schools with a stable organization for all of the recommendations compared to the analysis including all schools (even though no statistical significance was observed for all but one of the indicators). Our findings are in line with the literature demonstrating that implementation strategies are more likely to succeed in a stable organization, and schools are no exception [32, 36]. At the same time, leadership has been shown to have an important role for successful implementation [32]. The unplanned organizational changes observed in the present study could also explain why the magnitude of the change decreased for nearly all recommendations from 6 to 12 months. Achieving sustainment is a common challenge in intervention studies, including the ones in the education sector [36]. Sustainability can be influenced by many factors at different levels, such as principal support, personnel commitment, school climate, and organizational stability [36], to name just a few. Our findings underscore the importance of collecting information on potential contextual factors that may influence the implementation efforts in schools [37] and considering them in the analyses. One of the ways to prevent the negative effects of organizational instability on implementation is to ensure continuity within the implementation team, for example, by arranging for a substitute implementation team member.

### International relevance

Given that organizational and social risk factors for mental ill-health are a common challenge in school work environments worldwide [35, 38], results of this study are relevant in a broader international context. Despite some national variations, school settings share similar occupational demands such as high workload, work pace, emotional demands, and role conflicts, leading to increased stress, burnout, and sick-leave among school personnel [1, 3, 35, 39]. These features of school work environment are also the main factors behind turnover decisions of the school personnel [40]. Provided that many countries are facing large teacher shortages in the coming years [41], tackling organizational and social risk factors in the school environment to ensure teacher well-being and retention becomes an urgent policy issue in many national education systems.

As most of implementation effectiveness studies to date have been conducted in health care, and more research is needed on the effectiveness of implementation strategies in schools [31, 42]. Moreover, the majority of school-based implementation studies have often focused on student’s mental health and not school personnel’s; yet, successful implementation of the programs targeting student mental health is heavily dependent on the efforts of well-functioning school personnel [37, 43, 44]. This study has provided valuable knowledge regarding the effectiveness of a multifaceted strategy for the implementation of the guideline for the prevention of mental ill-health among the school personnel in the Swedish school setting, which can encourage studies in other countries to continue narrowing yet another, geographical, gap in school-based implementation studies [31, 42].

Finally, with the Swedish school setting sharing many similar features with other national school contexts, the knowledge on the effectiveness of the implementation strategies gained in the present study can be generalized to the school systems outside of Sweden [31]. Still, more controlled studies are needed, both in Swedish and other national school settings, that compare different implementation strategies aimed at prospectively identified barriers and facilitators to further our understanding of the effectiveness of implementation strategies on the relevant outcomes [31, 45, 46].

### Methodological considerations

Schools are still under-investigated settings when it comes to implementation research [31]. To our knowledge, this is one of the first implementation effectiveness studies aimed at prevention of mental ill-health of personnel conducted in the school setting. Further, the implementation strategies employed in this study were developed in a systematic way on the basis of the prospectively identified barriers and facilitators. The study design, with a cluster randomized controlled trial with parallel groups, is yet another strong feature.

A limitation of the study was that guideline adherence indicators developed by the research team were not tested for their psychometric properties. No validated instrument to assess the outcome measure of adherence to guideline in the school setting was found in the published literature. On the other hand, the outcome measure of adherence in this study was developed in a systematic manner, assessing specific target behaviors relevant to the guideline recommendations. Future studies should examine which measures would yield the most accurate assessment of guideline adherence in the school setting. One example of a more precise measure is the written occupational, safety, and health documentation collected from the school principals. Such written documentation can be accompanied by a corresponding checklist stating, for example, when the document was developed, which updates were made. Such documentation has previously been shown to be lacking in schools according to Swedish Work Environment Authority.

Further, more studies are needed to test the reliability and validity of the instrument assessing adherence to guideline recommendations. One of the ways to improve the measure employed in the current study is through the cognitive interview process—an important step of the instrument refinement to ensure that respondents interpret the questions as intended by the researchers [47]. Another alternative is to combine the implementation outcome measure used in the present study with the written documentation collected from the schools.

Finally, analysis for some of the guideline adherence indicators may have been underpowered to reveal statistically significant intervention effects. As the sample size was considerably smaller for the adherence indicators of recommendation 3, due to a preceding filter question, these results should be interpreted with caution. However, for most of the guideline adherence indicators, confidence intervals point to the possibility of significant effects should the study be replicated on a larger sample of school personnel. Ultimately, aiming at the practical significance over the statistical one may be particularly relevant for this study [48, 49], which is one of the first to assess implementation effectiveness of the guideline for prevention of work-related mental ill-health in the Swedish school setting.

## Conclusions

This study compared implementation effectiveness between two strategies of the guideline for mental ill-health at the workplace, in Swedish schools. The conclusion of the study is that the multifaceted strategy was not more effective than the single strategy in improving guideline adherence. Clearly, more work needs to be done on further development and validation of the guideline adherence instrument to be used in the school setting, both in Sweden and internationally. Future research is also needed on how to complement and sustain a positive impact of a multifaceted implementation strategy. This will certainly aid the efforts to implement guideline recommendations and thus support school personnel’s well-being. In an upcoming study, we will add strategies to the multifaceted implementation strategy and test their implementation mechanisms in a broader range of Swedish schools to further our understanding of how exactly they affect guideline implementation.

## Supplementary Information

**Additional file 1. **The TIDieR (Template for Intervention Description and Replication) Checklist*.

**Additional file 2. **CONSORT 2010 checklist of information to include when reporting a randomised trial*.

## Data Availability

The datasets generated and/or analyzed during the current study are not publicly available as the ethical approval for the study does not permit data sharing but are available from the corresponding author on reasonable request.
